# Gaseous ammonia pretreatment lowers the required energy input for fine milling-enhanced enzymatic saccharification of switchgrass

**DOI:** 10.1186/s13068-015-0315-y

**Published:** 2015-09-15

**Authors:** Bruce A. Diner, Jelena Lasio, Carl E. Camp, H. David Rosenfeld, Janine Fan, Bradley C. Fox

**Affiliations:** Industrial Biosciences, E. I. du Pont de Nemours and Co., Experimental Station, 200 Powder Mill Road, Wilmington, DE 19803 USA; Chemours Titanium Technologies, DuPont Experimental Station, 200 Powder Mill Road, Wilmington, DE 19803 USA; Central Research and Development, E. I. du Pont de Nemours and Co., Experimental Station, 200 Powder Mill Road, Wilmington, DE 19803 USA

**Keywords:** Lignocellulosic biomass, Pretreatment, Ball milling, Attritor milling, Gaseous ammonia, Switchgrass

## Abstract

**Background:**

Fine milling of dry lignocellulosic biomass, without prior chemical pretreatment, can produce a high percent theoretical yield of sugars during subsequent enzymatic saccharification. However, the high sugar yields, necessary for a commercial biofuels process, are costly, with the milling energy input, necessary to achieve such yields even exceeding the energy content of the biomass. In this study, we show that low moisture gaseous ammonia pretreatment of switchgrass, in advance of the milling step, significantly reduces the milling energy required to give high sugar titers.

**Results:**

We have found that the increase in monomeric sugar yields upon enzymatic saccharification of ball-milled, but not chemically treated switchgrass, is more closely tied to the formation of crystallites of cellulose with a negative linear dependence on the coherent domain size than to a decrease in particle size or to an increase in surface area of the biomass. The milling energy required to reach ~80 % of theoretical yield of glucose under these conditions is intolerably high, however, approximating two times the energy content of the biomass. Two different low moisture content ammonia pretreatments, prior to milling, significantly reduce the required milling energy (four to eightfold, depending on the pretreatment). These involve either heating the biomass at 150–160 °C for 1 h at 10 wt% gaseous ammonia or incubating at room temperature for 9 days at 20 wt% gaseous ammonia, the latter mimicking potential treatment during biomass storage. We have tested this combination of pretreatment and milling on switchgrass using a variety of milling methods, but mostly using ball and attritor milling. In the case of the high-temperature gaseous ammonia treatment followed by attritor milling, the increase in the monomeric sugar yield upon saccharification shows a negative linear dependence on the second or third power of the cellulose crystalline coherent domain size, implying that the surfaces as well as the ends of the cellulose fibrils are accessible to cellulolytic enzymes.

**Conclusions:**

The combination of knife milling, low moisture gaseous ammonia pretreatment followed by attritor milling that costs only ~5 % of the energy content of the biomass for a total energy input of ~11 % of the biomass energy content, is capable of delivering high sugar titers upon enzymatic saccharification. These results show, therefore, how to better integrate a mechanochemical step into the pretreatment of switchgrass in a commercial biomass to biofuels conversion process.

## Background

Exploitation of lignocellulosic biomass for biofuels can play a significant role in meeting the transportation energy needs of the United States. It is abundant, shows a positive energy balance for ethanol production [1], includes agricultural waste that avoids the competing interests of fuel vs. food [2], and can provide up to 30 % of US fuels in an environmentally sustainable manner [3]. Lignocellulose as is, however, is resistant to both chemical and enzymatic hydrolysis. In the case of enzymatic hydrolysis, a pretreatment step is required to render the lignocellulosic polysaccharides more accessible to cellulases and hemicellulases [4]. The most significant factors limiting enzyme efficacy are thought to be lignin content, cellulose crystallinity and xylan acetylation [5]. Here we will provide evidence for a synergistic interaction of fine milling preceded by chemical pretreatment and the impact that they have on enzymatic saccharification.

Some form of size reduction is a necessary first step toward conversion of lignocellulosic biomass to liquid fuels. Milling (e.g., knife and hammer milling) makes biomass handling easier and the material more susceptible to chemical pretreatment, facilitating enzymatic hydrolysis of polysaccharides. The efficiency of a milling technique will depend on the technique’s total specific energy for a given screen size, as well as proper matching of the biomass type (sensitivity to tensile and shear stresses) to the milling method that is the most effective [6–8]. Fine milling (e.g., ball milling, attritor milling), in addition to increasing the particle surface to volume ratio, increasing cellulase accessibility [9], can result in a significant reduction in the degree of polymerization, of both cellulose [10, 11] and lignin [12, 13], as well as a reduction in the crystallinity of cellulose [10, 14], all of which aid in enzymatic sugar release and subsequent sugar fermentation. Milling energy demand increases rapidly with reduced particle size [7, 15], so that the energy requirements for size reduction can increase to the point where they outweigh the benefits, expressed in terms of efficient saccharification and fermentation, making the overall process energetically unfavorable [16]. To reduce the energy required for mechanical pretreatment of biomass [17] or to enhance the saccharifiability of milled biomass [18], a chemical pretreatment can be used either preceding or coincident with mechanical pretreatment. Additional examples of such synergy, with particular attention to the energy efficiency of the combined processes, have been provided by Zhu [19, 20] and Barakat [21, 22] and their coworkers.

We have developed a lignocellulosic biomass pretreatment technology that applies low moisture content gaseous ammonia pretreatment followed by fine milling. Shown here for switchgrass, the gaseous ammonia pretreatment greatly reduces the energy required for milling and results in excellent sugar yields upon saccharification. It is expected that this technology would be applicable to a variety of lignocellulosic feedstocks.

## Results and discussion

### Ball milling of untreated switchgrass

The experimental work that follows was conducted on several batches of dry (>90 % DM) spring- and fall-harvested switchgrass obtained from Genera Energy (Vonore, TN, USA). These were knife-milled through a 1-mm screen and analyzed for carbohydrate and lignin as described in the “Methods” section. The samples were stored at room temperature until use. Their compositional analyses are shown in Table 1.

**Table 1:** Composition of switchgrass samples

Lot	UT-2 untreated (%)	UT-4a untreated (%)	UT-4b untreated (%)	UT-4b after 150–160 °C, 1 h with 10 wt% NH_3_ (%)	UT-5 untreated (%)
Glucan	37.61 ± 1.43	36.29 ± 0.19	34.10 ± 0.62	33.76 ± 0.38	35.95 ± 0.96
Xylan	25.67 ± 1.50	22.75 ± 0.95	20.52 ± 0.35	20.86 ± 0.20	24.53 ± 0.28
Arabinan	3.00 ± 0.12	2.77 ± 0.10	2.75 ± 0.19	3.03 ± 0.19	2.92 ± 0.10
Lignin	23.4	19.5	17.6	18.29	18.3

Compositional analyses of treated and untreated switchgrass samples were carried out as described in the “Methods” section and are expressed as percent of dry matter. The variation is the standard deviation of three or more measurements

**Fig. 1:**
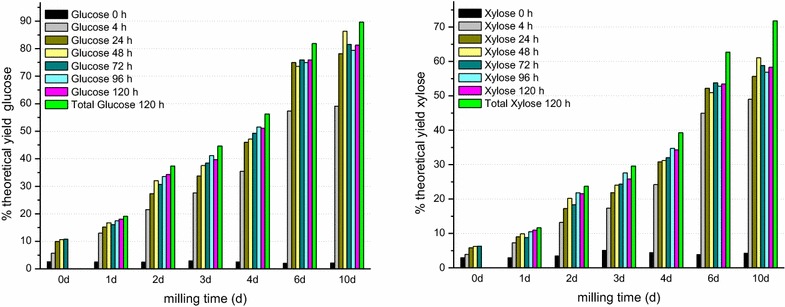
Monomeric glucose (*left*) and xylose (*right*) saccharification yields as a function of milling time. Five grams of UT-2 spring-harvested 1-mm knife-milled switchgrass was milled at room temperature using 200 g of 0.25″ stainless steel beads in a 125-mL plastic bottle at an end-to-end rotation speed of 83 rotations/min. Saccharification was performed for the indicated times (in hours) in 50 mM citrate buffer, pH 4.9 with Accellerase 1500 (25 mg protein/g glucan) + a beta xylosidase cocktail (16.6 mg protein/g xylan) at 47 °C using a 14 % solids loading. At 120 h, of saccharification, both the monomeric and the monomeric plus oligomeric (total) sugar yields are indicated

Guerra and coworkers [23, 24] have described a procedure (EMAL) for lignin isolation that involves extensive ball milling followed by mild acid hydrolysis. The first step in this procedure is a ten-day-long milling procedure, followed by enzymatic hydrolysis. We have found that this lengthy milling process renders switchgrass highly susceptible to enzymatic saccharification. Compared to untreated spring-harvested switchgrass (UT-2) that had been knife-milled through a 1-mm screen, a further 10 days of ball milling, showed an eightfold increase in monomeric glucose yields, totaling 81 % of theoretical yield upon saccharification for glucose monomer and 90 % of theoretical yield for soluble glucose monomer plus oligomer (Fig. 1). The corresponding yields for xylose monomer and total soluble xylose monomer plus oligomer were 58 and 72 %, respectively. The ability to attain such high saccharification yields without chemical pretreatment has distinct environmental advantages and was the starting point for an effort to further understand the reasons for the enhanced sugar accessibility and its applicability to commercial processing of lignocellulosic biomass to ethanol.

#### Particle size and surface area

**Fig. 2:**
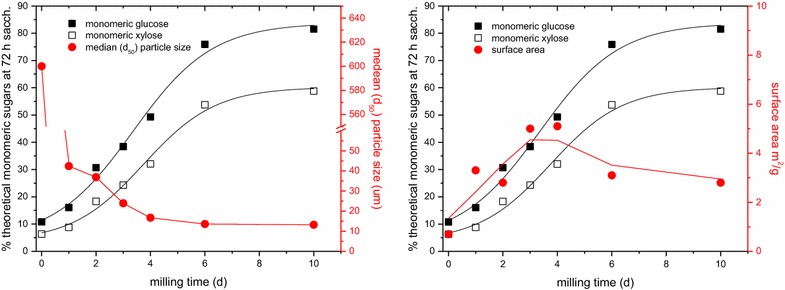
*Left* monomeric glucose and xylose yields at 72 h of saccharification of ball-milled switchgrass from Fig. 1 compared to the median particle size as a function of the ball milling time. Saccharification was carried out as in Fig. 1. *Right* monomeric glucose and xylose yields at 72 h of saccharification of ball-milled switchgrass from Fig. 1 compared to the surface area as a function of the ball milling time. Saccharification was carried out as in Fig. 1

To better understand the physical changes that occur during mechanical pretreatment, we measured the particle size of the milled material, to see whether the increase in saccharification yields followed the reduction in particle size (Fig. 2, left; Table 2). The particle size dropped from a median particle size (*d*_50_) of ~600 μm following knife milling to 16.7 μm after 4 days of ball milling, with very little further change in particle size upon additional milling. In fact, in some cases even a small increase in particle size was observed with further milling (vide infra), presumably due to aggregation. The saccharification yields, however, continued to rise another 100 % upon continued milling. Measurements of the surface area using N_2_ gas adsorption (Fig. 2, right; Table 2) showed a peak in the surface area per gram between 3 and 4 days at ~5 m^2^/g, decreasing to 3 m^2^/g with additional milling. The decrease is likely due to a collapse of the pore structure within the biomass. Both of these measurements show in the later stages of milling little correspondence between saccharification yield on the one hand and particle size and total surface area on the other. Nonetheless, it is likely that they contribute somewhat to enzyme accessibility in the earlier stages of milling.

**Table 2:** Particle size, surface area, and crystallinity of milled and unmilled switchgrass samples

Sample	Particle size (*d*_50_, µm)	Surface area (m^2^/g)	CrI (%)	Coherent domain size (nm)
KM UT-2 no pretreatment	600	1.34473	62.9	3.1
Plus ball milling 1 day	42.4	2.42792	62.7	3.4
Ball milling 2 days	36.9	3.56033	56.3	3.1
Ball milling 3 days	23.9	4.54339	48.2	2.7
Ball milling 4 days	16.7	4.52749	32.3	2.3
Ball milling 6 days	13.57	3.51319		1.4
Ball milling 10 days	13.2	2.94745		1.3
KM UT-4b no pretreatment	600	ND	61.1 ± 1.9	3.23 ± 0.15
KM UT-4b treated with gaseous NH_3_, 150–160 °C, 1 h	600	ND	71.0 ± 3.9	3.95 ± 0.07
Plus 5 min in Union Process SD-1 attritor mill	30.2	ND	59.5	3.5
Attritor milling for 10 min	19.0	ND	55.4	3.1
Attritor milling for 15 min	17.8	ND	50.4	3.0
Attritor milling for 20 min	13.55	ND	32.0	2.7
Attritor milling for 30 min	16.4	ND	21.6	2.2
Attritor milling for 60 min	18.9	ND	2.2	1.7
NH_3_-treated KM UT-4b plus 2 passages through Kemutec 5H Universal Pin Mill	84	ND	73.6	3.9
NH_3_-treated KM UT-4b plus jet milling in Fluid Energy Model 4 Microjet	23.4	ND	70.0	3.8
NH_3_-treated KM UT-4b plus passage through Hosokowa 2ACM Air-classifier mill	22.03	ND	66.7	3.5

Particle size, surface area, crystallinity index (CrI) and coherent domain size of 1-mm knife-milled (KM) switchgrass subjected to further fine milling with and without 150–160 °C, 1 h, 10 wt% ammonia pretreatment. The variation is the standard deviation of two measurements

#### Crystallinity

X-ray powder diffraction was used to characterize the crystallinity of the cellulose in the milled samples. Background subtraction and determination of the so-called crystallinity index (CrI, Table 2) were carried out according to methods described by Segal [25]. This method relies on a ratio of diffracted intensities at two specific two-theta angles to establish a measure of relative crystallinity. A number of authors have attempted to improve on this method. It is desirable to have a more absolute measure of crystallinity, and to allow for swelling of the cellulose lattice, changes in crystallite (coherent domain) size and two-theta zero offset errors. Bansal [26] describes a multivariate approach that involves fitting the diffraction pattern as a linear combination of model patterns representing crystalline and amorphous standards. Thygesen [27] describes a modified Rietveld refinement which fits the crystalline contribution to the diffraction with a calculated pattern based on the known crystal structure of alpha cellulose and a fitted background representing the amorphous contribution. In either of these methods, the crystalline index is the ratio of the area under the crystalline model to the area under the whole diffraction pattern. The Rietveld method has the advantage of allowing for changes in the crystalline lattice. Both swelling from absorption of solvents and reduction of crystallite size can be accommodated and extracted from the modeling as useful data points in and of themselves.

We utilize a hybrid method in which the Rietveld refinement is carried out on a limited range of two-theta data (10°–32°) which has had a linear background subtracted from it as in the Segal method. We base our structural model on the known crystalline structure of alpha cellulose [28]. Rather than fit an arbitrary function to represent the amorphous contribution, we fit a linearly scaled pattern from a fully amorphous standard. To reduce the number of parameters in our refinement, we do not fit the interaxis angles or the *c*-axis of the crystalline lattice. The *a* and *b* axes are only fit when crystallinity and coherent domain size are sufficiently large to allow clear resolution of the crystalline peaks. Our reported crystalline fraction is the ratio of the area under the Rietveld refined crystalline model to the area under the fitted range. Coherent domain size is extracted from crystalline peak breadths obtained from the refinement [29] and is the average distance over which the crystalline cellulose is free of structural defects.

**Fig. 3:**
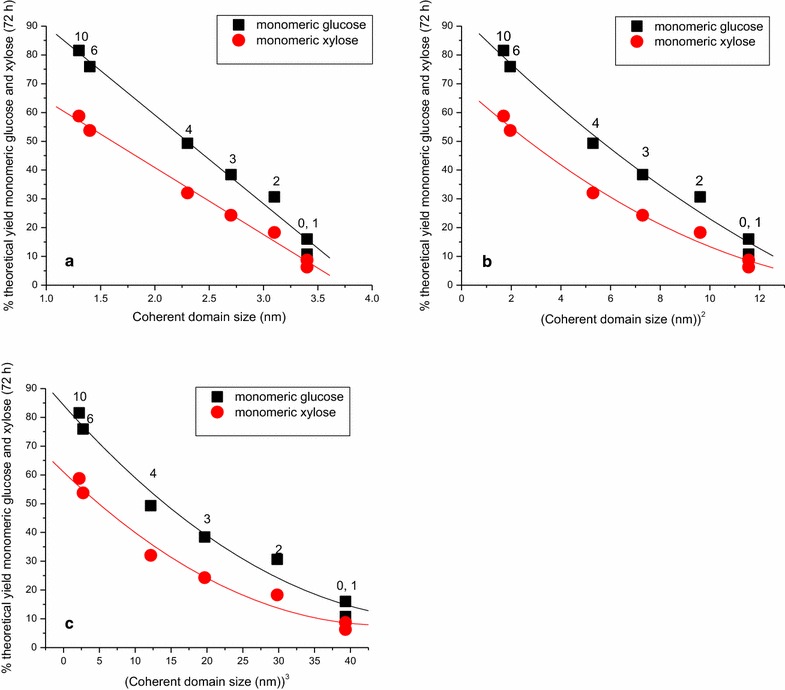
**a**, **b** and **c** Monomeric glucose and xylose yields at 72 h of enzymatic saccharification of ball-milled switchgrass from Fig. 1 compared to the coherent domain size determined by wide-angle X-ray diffraction. In figures **a**, **b** and **c** the coherent domain size is plotted to the first, second and third powers, respectively. The numbers above the *plotted points* are the ball milling times in days. Saccharification was carried out as in Fig. 1. The *size of the symbols* are representative of the standard deviations on both axes

Figure 3 shows plots of the coherent domain size (see also Table 2), extracted from the X-ray diffraction data at different ball milling times, to the first, second and third powers against the monomeric sugar yields obtained following 72 h of enzymatic saccharification. The plot of the coherent domain size versus the monomeric sugar yields shows a much more direct correlation between milling and saccharification yield than was observed for particle size and surface area. That the 72-h monomeric sugar yield shows a negative linear relationship to the first power of the coherent domain size, suggests that the enzymatic digestion occurs in a one-dimensional fashion and primarily at the ends of the cellulose fibrils, exposed by ball milling. The same appears to be true for hemicellulose. The one-dimensional character of the digestion implies that surface accessibility by cellulases to the cellulose fibrils is likely blocked by hemicellulose and lignin, in these milled but otherwise untreated samples. Likewise, surface accessibility by xylanases is probably blocked by lignin.

While the negative linear correlation of the saccharification yields to the coherent domain size observed here implies a direct cause and effect relationship between them, it is possible that there are other mechanochemical changes that occur in parallel with the changes in cellulose crystallinity. For example, the formation of crystallites, as we are implying in the present case (see also Garvey et al. [30] and Park et al. [31]), may be accompanied by a decrease in the degree of polymerization of cellulose by homolytic bond cleavage of 1,4-glycosidic bonds [32–34] or a cause of it. Similar bond cleavage may well be occurring in hemicellulose and in lignin [13], both of which could contribute to increased enzyme accessibility to polysaccharides.

In studies of biomass composition, Ralph and coworkers have reported that upon vigorous planetary ball milling, the degree of polymerization of cellulose decreased from 7 to 10,000 in the plant cell walls to ~25 in milled material [11, 35]. Ball milling also homolytically cleaves C_α_–C_β_ carbon–carbon bonds of the propionate chain and C_β_–O ether linkages of lignin [12, 13]. Hydroxy radicals that form in the process do further lignin oxidation. Therefore, even though no chemical is used in the milling step, the material is chemically altered. What is particularly attractive about mechanical pretreatment is that the high saccharification yields generated upon enzymatic saccharification occur in the absence of a chemical pretreatment and with no removal of lignin. The latter is generally considered to be a barrier to enzyme diffusion within the biomass and a surface onto which enzyme molecules adhere ineffectively [36]. As a consequence, many pretreatments are aimed at modifying or extracting lignin [37]. The elevated saccharification yields obtained in the experiments with mechanical pretreatment alone are consistent with the view that significant lowering of the crystallinity index is sufficient to enhance enzymatic hydrolysis regardless of the lignin content [5], obviating the cost and additional unit operations associated with lignin extraction.

Conventional ball milling comes, however, at considerable energy cost. The power, *P*, required to operate a ball mill, exclusive of mechanical losses in the motor and gearbox (an additional 5–10 %), is approximated by the following equation: *P* = 0.285*d*(1.073 − *j*)*mn*, where *d* is the internal diameter of the milling vessel in meters, *j* is the fraction of the milling vessel occupied by the milling media, *m* is the total mass in tons of the media within the mill and *n* is the milling speed in rpm. Applying this equation to the bottles, media and loading used for ball milling (see “Methods” section) an energy input equal to the energy content of the biomass (9.1 × 10^4^ J/5 g) [38, 39] is consumed within 5.4 days. If one includes an additional 10 % due to mechanical losses, then this amount of energy is consumed in 4.9 days of milling. Five days of ball milling of spring-harvested switchgrass, yields about 60 % of the theoretical yield of monomeric glucose and about 42 % that of monomeric xylose upon 72 h saccharification (Fig. 1), making ball milling alone far too costly as a pretreatment process.

### Milling of pretreated switchgrass

#### Combination of gaseous ammonia and ball milling

In light of the high energy cost of extensive ball milling, we sought ways to decrease the amount of energy required to mill the biomass. We [40] as well as a number of other authors [17–19, 21] have advocated for chemical pretreatment as a means to decrease the amount of energy needed in simultaneous or subsequent mechanical milling. The chemical pretreatment is also preferentially done at a low liquid to solids ratio (*L*/*S* w/w) to decrease the energy required to heat and vaporize any water that is present [20, 22, 40]. We ultimately focused on pretreating the biomass with gaseous ammonia, either at elevated or ambient temperature. The advantages of using ammonia in a step preceding milling are several fold: ammonia breaks ferulate ester bonds to hemicellulose, diminishing biomass cross-linking, and cleaves acetyl ester bonds of hemicellulose, improving the pentose sugar yield upon subsequent enzymatic sacharification [41]. It also allows for a more favorable acetamide/acetic acid ratio than the processes that involve more water (acetamide is less inhibitory than acetic acid to the ethanologen). Also, the use of gaseous ammonia leaves biomass dry, when ammonia is administered and flashed off at high temperature. The biomass is left with a dry matter (DM) content of >90 % consistent with an optimum of 90–93 % DM for ball milling [42]. Finally, any unreacted ammonia can be readily recycled to be used again and the use of ammonia means that there are no inorganic salts left to be disposed of at the end of the process. Note as well (Table 1, UT-4b) that the 160 °C ammonia pretreatment does not modify the polysaccharide composition of the biomass.

**Fig. 4:**
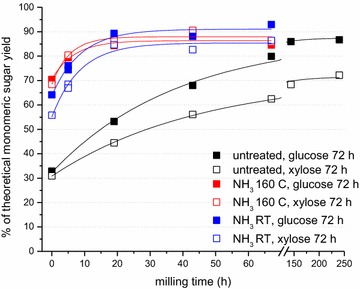
Monomeric sugar yields as a function of ball milling time with ¼″ stainless steel beads for 1-mm knife-milled untreated and treated UT-4a switchgrass (10 wt% gaseous NH_3_, 160 °C, 1 h) and 1-mm hammer-milled UT-4a switchgrass treated (20 wt% gaseous NH_3_) at room temperature, 9 days and then 1-mm knife-milled. The data points are fit with exponential rise curves. Saccharification for 72 h was carried out as in Fig. 1 except that the buffer concentration was 100 mM Na citrate, pH 4.9 and enzyme concentrations were 28 mg Accellerase^®^ 1500/g glucan and 21.5 mg beta xylosidase cocktail/g xylan at a solids loading of 14 %

Fall-harvested switchgrass (UT-4a) was knife-milled through a 1-mm screen and subjected to 10 wt% gaseous ammonia treatment at 160 °C with a 1-h hold time (see “Methods”). Another sample (1-mm hammer milled) was incubated at room temperature with 20 wt% ammonia for 9 days and then knife-milled through a 1-mm screen. Following the ammonia pretreatments, the ammonia was flashed off leaving a dry sample (initially 98 and 92 % DM, respectively). These ammonia-treated samples were subsequently ball-milled with 1/4″ spherical steel beads from 0 to 67 h and compared to a similarly ball-milled but untreated switchgrass sample (91 % DM, initially 1 mm knife-milled). The moisture content of the ammonia-treated samples increased slightly during milling, ultimately reaching 94 % DM (for the 160 °C, 1 h sample) and 91 % DM (for the room temperature 9 days sample) after 67 h of milling. All three samples were then saccharified at 14 % solids loading. Figure 4 shows that the ammonia pretreatments greatly enhanced the efficacy of milling on the saccharification yields compared to the untreated sample. Relative to the untreated control, the 160 °C, 1 h and room temperature, 9-day ammonia-pretreated samples showed *t*_1/2_ exponential rise times—5.7 and 4.4-fold faster, respectively, for glucose and 8.4 and 5.3-fold faster, respectively, for xylose (Table 3).

**Table 3:** Kinetics of increase in monomeric sugar yields as a function of milling time

Switchgrass sample	Sugar	Rise time (*t*_1/2_) (h)
Untreated	Glucose	26.8
Untreated	Xylose	31.4
10 % NH_3_, 160 °C, 1 h	Glucose	4.66
10 % NH_3_, 160 °C, 1 h	Xylose	3.75
20 % NH_3_, RT, 9 days	Glucose	6.11
20 % NH_3_, RT, 9 days	Xylose	5.95

Exponential rise times (*t*_1/2_) in 72-h saccharification yields as a function of ball milling time for gaseous ammonia-treated (160 °C and room temperature) and untreated UT-4a switchgrass (from Fig. 4)

The ammonia-treated samples, in contrast to the untreated sample, show slightly faster rise times for xylose than for glucose and glucose and xylose yields that are more similar and higher than for the untreated control. These phenomena likely arise from the hydrolysis and ammonolysis of acetyl ester and feruloyl ester bonds to xylan and from partial defragmentation of lignin. Deacetylation and breakage of feruloyl ester bonds increase the accessibility of xylan to xylanases, while scission of feruloyl ester bonds and defragmentation of lignin decrease the extent of cross-linking of lignin with xylan and with itself, increasing access of all cellulases and hemicellulases to polysaccharide and weakening the structural integrity of the biomass.

As switchgrass cannot be harvested throughout the year, a means of stable storage of this material is required prior to biofuel conversion. Room temperature ammonia incubation has the advantage of providing an aseptic storage medium that doubles as a pretreatment. The similarity between the short high-temperature treatment and the long room temperature treatment implies that satisfactory ammonia treatment may be carried out under very mild conditions provided the incubation time is long enough.

#### Varied solid loadings

**Fig. 5:**
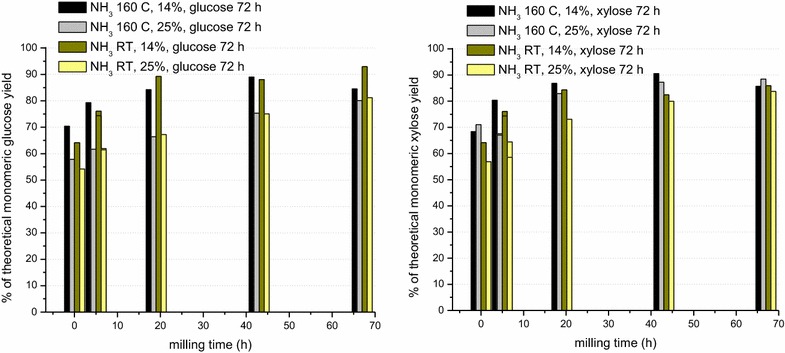
Comparison of monomeric glucose (*left*) and xylose (*right*) yields at 14 and 25 % solids loading after 72 h saccharification as a function of milling time using ¼″ stainless steel beads for the ammonia-treated samples of Fig. 4. Saccharification was carried out as in Fig. 4

Higher solid loadings during saccharification yield higher sugar and ethanol titers during saccharification and fermentation, respectively, and so have a positive impact on the economics of the overall process. Increased solids loading in saccharification, however, can be accompanied by difficulties in mixing and liquefaction, as well as by increased product inhibition of enzymatic hydrolysis due to higher product sugar concentrations. The ball milling treatment described above, however, provides satisfactory rheology such that the biomass can be readily stirred and liquefied even at solids loadings of 25 %. Figure 5 shows a comparison of the monomeric sugar saccharification yields at 72 h as a function of milling time for samples that were subsequently saccharified at 14 and 25 % solids loading. As the milling time increases for the 160 °C, 1-h sample, the difference in the percent monomeric yield for glucose decreases such that at 67 h of milling there is little difference between the two loadings. A more significant difference remains in the glucose yields for the room temperature, 9-day sample. In the case of the xylose yields at 72-h saccharification, there is little difference between the two solids loadings for the 160 °C, 1-h sample from 19 h of milling on. A more significant, but lesser difference than for glucose, remains for the xylose yields as the milling time increases for the room temperature sample. While the 160 °C treatment appears to be somewhat more aggressive, it is likely that the 160 °C and RT gaseous ammonia-treated samples are likely to converge even further in their milling and saccharification behavior as the room temperature incubation time for the latter increases.

#### Attritor milling

Attritor milling differs from ball milling in that the chamber containing the milling medium is stationary and the medium is displaced by a rotating shaft with side arms. According to Szegvari and Yang [43], attritor milling uses about half as much energy as conventional ball milling to reach the same median size in the milling of chalcopyrite. To evaluate attritor milling as a means to further reduce the milling energy, we performed a series of milling experiments with an attritor mill using 1-mm knife-milled fall-harvested UT-4b switchgrass previously pretreated with 10 % gaseous ammonia at 150–160 °C for 1 h (98 % DM). An SD-1 Union Process attritor mill, with L-arms attached to the rotating shaft, was loaded with 500 g of pretreated switchgrass and 40 lbs of ¼″ stainless steel beads. The mill was run at 516 rpm (2.14 hp) with the jacket cooled by running tap water. Time points were taken periodically up to 1 h. The energy input of the attritor mill under these conditions was such that 5 min of milling [0.958 MJ/kg (0.266 kW-h/kg)] corresponded to about 5 % of the energy content of the switchgrass [18.22 MJ/kg (5.06 kW-h/kg)] [38, 39].

**Fig. 6:**
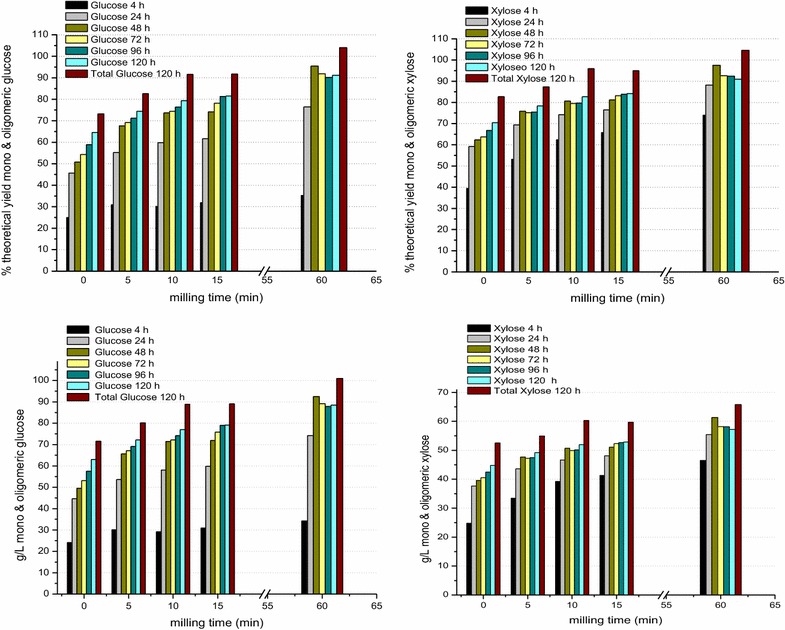
Percent of theoretical sugar yields (*top*) and concentration of sugars (*bottom*) as a function of attritor milling time for glucose (*left*) and xylose (*right*) monomers and oligomers. Knife-milled (1 mm) fall-harvested UT-4b switchgrass was treated with gaseous ammonia at 150–160 °C for 1 h and milled in an attritor mill for 5, 10, 15 and 60 min (1/4″ spherical stainless steel beads, 40 lbs./500 g biomass). The samples were then saccharified using an enzyme consortium, Accellerase^®^ DUET at 28 mg protein/g glucan + xylan for the indicated times. The unmilled sample was saccharified using an enzyme consortium with the same components as Accellerase^®^ DUET. This formulation gave the same saccharification yields for glucose and xylose in the milled samples, within 5 % absolute, as the actual Accellerase^®^ DUET All yields are monomeric except for the brown bar which is the total soluble sugar (monomer + oligomer) at 120 h of saccharification. The saccharification was performed at a solids loading of 25 %

Saccharification trials were run at 25 % solids loading on the attritor-milled samples using Accellerase^®^ DUET [28 mg enzyme/g (glucan + xylan)]. Figure 6 shows, as a function of milling time, the saccharification yields and titers [percent of theoretical yield (top) and sugar concentration (bottom), respectively] for monomeric glucose and xylose at 24-h saccharification intervals and for total soluble glucose and xylose after 120 h of saccharification. After 5 min of attritor milling, the monomeric sugar yields at 72 h of saccharification at 25 % solids loading, 69 and 75 % for glucose and xylose, respectively, are similar to the yields observed at 72 h of saccharification for the 160 °C, 1-h ammonia-treated 20-h ball-milled sample of Fig. 5 at 25 % solids loading (67 % glucose and 83 % xylose). This comparison shows that the attritor mill takes 120 to 240-fold less time to mill gaseous ammonia-treated switchgrass as compared to the conditions used for the conventional ball mill, consistent with their respective inputs of kinetic energy—0.958 MJ/kg biomass in 5 min for the attritor mill, 1.397–2.794 MJ/kg in 10–20 h for the conventional ball mill and a twofold higher efficiency for attritor milling over ball milling [43]. The total soluble sugar yields at 120 h of enzymatic saccharification were ~100 % of theoretical for both glucose and xylose after 60 min of attritor milling. The total soluble sugar titers (Fig. 6) after 120 h of saccharification correspond to a total of 135 g (glucose + xylose)/L for 5 min of attritor milling and 167 g (glucose + xylose)/L for 60 min of attritor milling. The total soluble sugar yields at 120 h were used to better simulate the sugar content attainable from 3 days of enzymatic saccharification followed by 2 days of fermentation with the enzymes still present.

#### Comparison of particle size, crystallinity and saccharification of attritor-milled gaseous ammonia-treated switchgrass

**Fig. 7:**
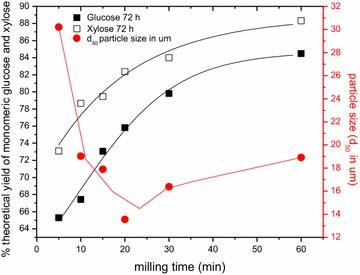
Comparison of the 72 h monomeric glucose and xylose saccharification yields from Fig. 6 with the *d*_50_ particle size, determined by light scatter, as a function of attritor milling time

Figure 7 shows, as in the case of the ball-milled untreated switchgrass (Fig. 2, left), that there is a poor correlation between the particle size and the monomeric sugar yields upon saccharification for gaseous ammonia-treated switchgrass (Fig. 7), except possibly early on in the attritor milling process. In fact, after 20–25 min of attritor milling the particle size (Fig. 7, Table 2) reaches a minimum and then increases with additional milling time, even though the saccharification yields continue to increase monotonically. It is likely that upon additional milling, the particles tend to aggregate, while decrystallization and depolymerization continue and have a beneficial effect on saccharification yields.

**Fig. 8:**
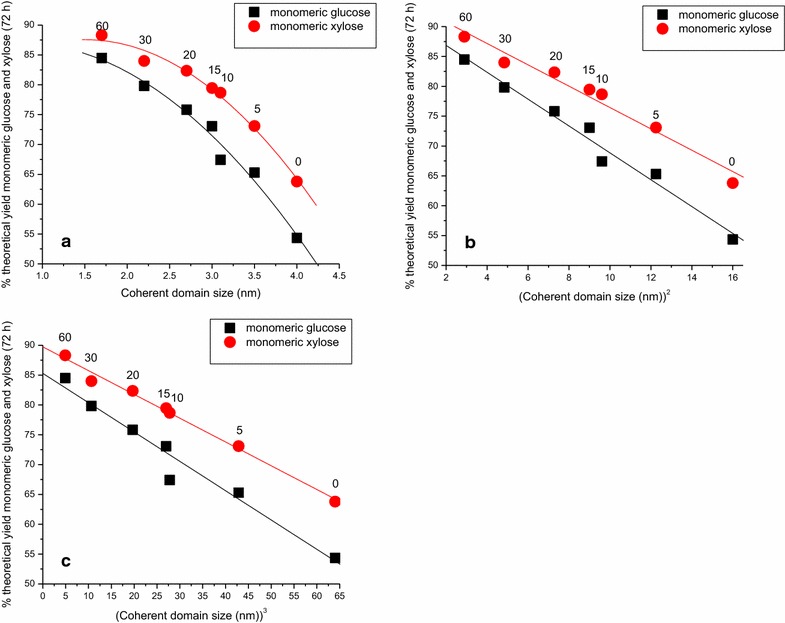
**a**, **b** and **c** Monomeric glucose and xylose yields at 72 h of enzymatic saccharification of gaseous ammonia and subsequently attritor-milled switchgrass from Fig. 6 compared to the coherent domain size determined by wide-angle X-ray diffraction. In figures **a**, **b** and **c**, the coherent domain size is plotted to the first, second and third powers, respectively. The *numbers* above the *plotted points* are the attritor milling times in hours. Saccharification was carried out as in Fig. 6. The *size of the symbols* are representative of the standard deviations on both axes

Figure 8 shows plots of the coherent domain size (see also Table 2), extracted from the X-ray diffraction data at different attritor milling times, to the first, second and third powers, versus the monomeric sugar yields obtained following 72 h of enzymatic saccharification. The crystallinity index and the coherent domain size in the absence of milling actually increase from 61 to 71 % and from 3.23 to 3.95 nm, respectively, as a consequence of the 150–160 °C gaseous ammonia pretreatment (Table 2), likely because some annealing of the crystalline phases occurs during chemical pretreatment. The extent to which the coherent domain size decreases upon either ball milling or attritor milling is not very different between the experiments shown in Figs. 3 and 8 (to 1.3 and 1.7 nm, respectively). However, unlike the negative linear dependence of the monomeric sugar yields, following 72 h of saccharification, on the first power of the coherent domain size, observed in Fig. 3 for milling in the absence of ammonia pretreatment, the sugar yields now show a negative linear dependence on the second and third powers of the coherent domain size. The higher power dependence suggests that the gaseous ammonia pretreatment combined with attritor milling has exposed the surfaces as well as the ends of the cellulose fibrils. The same appears to be true for hemicellulose. The gaseous ammonia treatment at 150–160 °C has cleaved the ferulate ester bonds linking the lignin to the hemicellulose as well as mobilizing the lignin [44], likely rendering both the surfaces and the ends of the cellulose fibrils and the hemicellulose more accessible to the cellulolytic and xylanolytic enzymes.

As in the earlier case of ball milling without pretreatment, it is likely that dislocations associated with the formation of crystallites are accompanied by or are a result of a decrease in the degree of polymerization of cellulose, hemicellulose and lignin [13, 32].

#### Other fine milling methods

Given the success in using gaseous ammonia treatment combined with attritor milling to increase the saccharification yields, we explored other fine milling methods to see if there were a milling technique that were both continuous and had even higher energy efficiency. Milling trials were conducted on a Kemutec 5H Universal Pin mill, a Fluid Energy Micro-Jet jet pulverizer and on a Hosokawa 2ACM air classifier mill. Gaseous ammonia-pretreated switchgrass was prepared as for attritor milling (Fig. 6, 1 mm knife-milled, 150–160 °C, 1 h) and subjected to the above-mentioned milling methods. All were milled in a continuous fashion with the jet pulverizer and air classifier mills provided with an automated continuous feed, enabling control over the energy input per unit weight of biomass. Given the scale of the milling trials, the pin mill had to be fed by hand, making it impossible to quantify the energy input per weight of biomass.

Table 4 shows the total soluble glucose and xylose yields after 120 h of saccharification for the different milling techniques compared to 5 min of attritor milling and to gaseous ammonia knife-milled switchgrass that had not been fine milled. Each of the samples was also saccharified with three different concentrations [7, 14 and 28 mg enzyme/g (glucan + xylan)] of Accellerase^®^ DUET at a 25 % solids loading. The energy inputs for the various techniques are as follows: Union Process SD-1 attritor mill (0.958 MJ/kg in 5 min), 2 passages of the Kemutec pin mill (energy input not determined), Fluid Energy Micro-Jet (5.40 MJ/kg) and Hosokawa 2ACM air classifier mill (1.801 MJ/kg). Of these the attritor mill run in batch mode provided the highest saccharification yields at the lowest enzyme loadings, and the lowest energy input. The next best method was the air classifier mill. While there are time savings associated with running a mill in continuous mode, attempts at running the attritor mill in continuous mode (Union Process HAS mill) gave lower saccharification yields at higher energy inputs than did the attritor mill run in batch mode.

**Table 4:** Saccharification yields, milling energy and energy efficiency of milled switchgrass samples at different enzyme loadings

Sample 7 mg enzyme/g (glucan + xylan)	Energy input during fine milling (MJ/kg)	Monomeric + oligomeric Glucose % theor. yield	Glucose mass yield (kg/kg biomass)	Energy efficiency (glucose mass yield/energy input) (MJ/kg)	Monomeric + oligomeric Xylose % theor. yield	Xylose mass yield (kg/kg biomass)	Energy efficiency (xylose mass yield/energy input) (MJ/kg)	Energy efficiency (glucose + xylose mass yield/energy input) (MJ/kg)
Union process attritor mill, 5 min	0.958	61.75	0.2334	0.244	81.13	0.1874	0.196	0.439
Hosokowa 2ACM Air-classifier mill	1.801	55.81	0.2110	0.117	74.56	0.1722	0.0957	0.213
Fluid energy model 4 microjet, jet mill	5.40	57.06	0.2157	0.0399	75.74	0.1750	0.0461	0.0886
Kemutec 5H universal pin mill, 2 passages	ND	53.57	0.2025		74.88	0.1730		
NH_3_-treated unmilled	NA	49.58	0.1874		59.61	0.1377		
Sample 14 mg enzyme/g (glucan + xylan)								
Attritor mill	0.958	81.77	0.3091	0.323	91.81	0.2121	0.221	0.544
Air-classifier mill	1.801	70.62	0.2669	0.148	83.18	0.1921	0.1068	0.255
Jet mill	5.40	66	0.2495	0.0462	74.11	0.1712	0.0319	0.0779
Pin mill, 2 passages	ND	59.69	0.2256		73.46	0.1697		
NH_3_-treated unmilled	NA	57.09	0.2158		63.07	0.1457		
Sample 28 mg enzyme/g (glucan + xylan)								
Attritor mill	0.958	82.57	0.3121	0.326	87.36	0.2018	0.211	0.537
Air-classifier mill	1.801	79.83	0.3018	0.168	87.65	0.2025	0.112	0.280
Jet mill	5.40	76.31	0.2885	0.0534	85.23	0.1969	0.0365	0.0899
Pin mill, 2 passages	ND	72.54	0.2742		79.36	0.1833		
NH_3_-treated unmilled	NA	61.89	0.2339		64.21	0.1483		

Saccharification yields and milling energies for milling trials on UT-4b NH_3_-treated (150–160 °C, 1 h) switchgrass. Each of the samples was saccharified at a 25 % solids loading for 120 h with three different concentrations of Accellerase^®^ DUET at 7, 14 and 28 mg protein/g (glucan + xylan). The sugar yields are based on soluble monomer plus oligomer

Table 4 also shows that the attritor-milled sample shows little difference in glucose yield between the 14 and 28 mg enzyme/g glucan + xylan enzyme loadings. In all of the other cases, there remains a significant increase in glucose yield in going from 14 to 28 mg/g (glucan + xylan). The more effective milling technology, therefore, significantly lowers the enzyme loading required for high saccharification yields.

#### Dependence of saccharification yields on moisture content during gaseous ammonia treatment

**Fig. 9:**
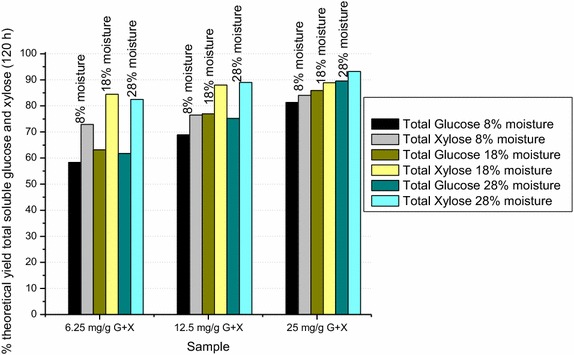
Comparison of saccharification yields as a function of the moisture content (8, 18 and 28 %) of UT-5 switchgrass during 10 wt% gaseous ammonia treatment (150–160 °C, 1 h). All of the UT-5 samples were attritor-milled for 8.5 min in a Union Process S-1 attritor mill. Each of the samples was saccharified at a 25 % solids loading for 120 h with three different concentrations of Accellerase^®^ DUET at 6.25, 12.5 and 25 mg protein/g (glucan + xylan). The saccharification yields are based on the total soluble monomer and oligomeric glucose and xylose

A head-to-head comparison (not shown) between 1-mm knife-milled switchgrass subjected to either 10 wt% dry gaseous ammonia (92 % DM final) treatment or to dilute aqueous ammonia pretreatment at 6 and 12 wt% ammonia (60–67 % DM final), all at 150–160 °C for 1 h, showed that the higher moisture content pretreatment gave a substantially higher xylose saccharification yield—63.1 % total soluble xylose for 10 wt% dry ammonia versus 72.7 and 82.6 % for 6 and 12 wt% dilute ammonia, respectively. The glucose yields were more similar. A likely explanation for this observation is that acetyl and feruloyl ester bond hydrolysis and ammonolysis are promoted by the higher moisture content pretreatment, making the xylan more accessible to enzymatic hydrolysis. To test if this was indeed so, we conducted a comparative study in which the moisture content of 1-mm knife-milled UT-5 switchgrass was adjusted to 8, 18 and 28 % prior to being placed in the 5-L Littleford Day horizontal pressure reactor for gaseous ammonia treatment. The water was added using an atomizer and the biomass was incubated overnight in a plastic bag at room temperature. These samples were then treated for 1 h at 150–160 °C with 10 wt% anhydrous ammonia. The samples had dry matter contents of approximately 98, 97 and 96 % after flashing off of the ammonia at the end of the gaseous ammonia treatment. The samples were milled for 8.5 min in a Union Process S-1 attritor mill, energetically equivalent to 5 min of milling in the Union Process SD-1 attritor mill. The moisture content of all three samples was low enough, following the gaseous ammonia treatment, to be compatible with attritor milling. The samples were then saccharified for 120 h at 47 °C at a solids loading of 25 % and at Accellerase^®^ DUET loadings of 6.25, 12.5 and 25 mg protein/g (glucan + xylan). Figure 9 shows that the 6.25 and 12.5 mg enzyme/g (glucan + xylan) enzyme loadings provide an absolute increase in xylose yield of 11.5 % each, for samples treated at 18 % as opposed to 8 % moisture content. Increasing the moisture content by another 10 % (28 % moisture content) had only a minor impact on the xylose yield relative to 18 % moisture content. There was also a small increase in the glucose yields. The 6.25 and 12.5 mg Accellerase^®^ DUET/g (glucan + xylan) enzyme loadings showed an absolute increase in glucose yield of 5 and 8 %, respectively, for samples treated at 18 % DM as opposed to 8 % moisture content. Increasing the moisture content by another 10 % (28 % moisture content) had only a minor impact on the glucose yield relative to 18 % moisture content. The larger increase in the xylose as opposed to the glucose yield is consistent with the initial hypothesis that the increased moisture content enhances cleavage of xylan ester linkages.

Fall and spring-harvested switchgrass typically differ in their moisture content, with a moisture content of around 35 % in the fall and 7 % in the spring [39]. Soluble sugars are lost over the winter and the percent lignin content increases. There is also a loss in harvestable biomass. All attest to the benefits of being able to use fall-harvested switchgrass. These results show that the gaseous ammonia pretreatment plus attritor milling can be performed on fall-harvested switchgrass despite the higher moisture content. Drying of the biomass, as a result of flashing off of the vapor phase at the end of the pretreatment (see above), should be sufficient for subsequent attritor milling.

### Energy considerations

Zhu [20] and Barakat [22] and their coworkers characterize pretreatment processes in terms of energy efficiency—the sugar yield (kg sugar/kg biomass) divided by the energy input necessary to attain that sugar yield (in MJ or kW-h/kg biomass), a useful analysis. Maximizing the energy efficiency for the production of fermentable sugars from lignocellulosic biomass is critical to a successful conversion process in terms of energy balance and cost. Also critical is generating the sugars in as concentrated a solution as possible such that the product of subsequent fermentation (e.g., ethanol) is generated in a concentrated form, requiring less energy to recover it from the fermentation broth. Table 5 shows a comparison of the energy inputs and energy efficiencies of the gaseous ammonia plus attritor milling process described here with processes that have been characterized with respect to energy input and efficiency in other laboratories. While the processes shown have not for the most part been carried out on switchgrass, some of them have been by some of the same authors (e.g., SPORL, dilute acid, alkali) [45] but without the determination of the energy input.

**Table 5:** Total energy input, saccharification yield and energy efficiency of grinding, chemical pretreatment and subsequent milling

Sample and pretreatment method	Total energy input of initial size reduction + chemical treatment + milling (MJ/kg)	Glucose mass yield (kg/kg biomass)	Energy efficiency (glucose mass yield/energy input, kg/MJ)	Other reducing sugars mass yield (kg/kg biomass)	Energy efficiency (other reducing sugar mass yield/energy input, kg/MJ)	Energy efficiency (total sugar mass yield/energy input, kg/MJ) [normalized to 65 % carbohydrate equivalent]
Switchgrass, 10 wt% gaseous ammonia + attritor milling^i^	2.012 (DM = 93 %)	0.309 (total soluble)^b^	0.154	0.242 (total soluble xylose + arabinose)	0.120 (xylose + arabinose)	0.274 [0.280] (total soluble sugars)
Switchgrass, extrusion and microwave treatment^a,j^	900 for microwave alone	0.199^c^	2.21 × 10^−4^	0.1744	0.194 × 10^−4^	4.14 × 10^−4^
Steam explosion (Spruce)^k^	1.977^f^ (L/S = 1)	0.346^d^ (monomeric)	0.175	h		[0.260] (monomeric sugars)
Organosolv (Lodgepole pine)^l^	2.687^f^ (L/S = 7) 1.613^f^ (L/S = 4) 1.254^f^ (L/S = 3)	0.347^e^ (monomeric)	0.129 0.215 0.277	h		[0.187] [0.311] [0.400] (monomeric sugars)
SPORL (Spruce)^m ^ (Lodgepole pine)^o^	1.863^f^ (L/S = 3) L/S = 3	0.372^d^ (monomeric)	0.200	h h		[0.350] (size reduction energy 150 kW-h/ton) (monomeric sugars) [0.270]
Wheat straw without chemical pretreatment^n^	2.149 ^g^ (L/S = 0.2)	0.118	0.0549	0.058		
Wheat straw with 5 % w/w NaOH dilute pretreatment^n^	2.867 ^g^ (L/S = 5)	0.332	0.116	0.181		
Wheat straw with 5 % w/w NaOH “dry” pretreatment^n^	1.307 ^g^ (L/S = 0.2)	0.32	0.245	0.212		
Wheat straw with 5 % w/w NH_3_ “dry” pretreatment^n^	1.620 ^g^ (L/S = 0.2)	0.14	0.0864	0.071		

The current process on switchgrass is compared to literature processes for which total energy inputs, saccharification yields and energy efficiencies have been calculated. The total energy input for all processes includes that required for grinding, chemical pretreatment and subsequent milling

^a^Polysaccharide composition assumed to be the same as in current paper

^b^Enzymatic hydrolysis at 25 % solids loading

^c^Enzymatic hydrolysis at 10 % solids loading

^d^Enzymatic hydrolysis at 2 % solids loading

^e^Enzymatic hydrolysis at 2 % cellulose loading

^f^Assumes thermal energy recovery of 50 % for thermal processes

^g^Energy calculations are net (power with biomass minus power without biomass) for centrifugal and ball milling

^h^Assumed to be recoverable from pretreatment stream

^i^Current work

^j^Karunanithy et al. [54]

^k^Söderström et al. [48] and Zhu et al. [20]

^l^Pan et al. [49] and Zhu et al. [20]

^m^Zhu et al. [20], SPORL (Sulfite Pretreatment to Overcome Recalcitrance of Lignocellulose)

^n^Barakat et al. [22]

^o^Zhu et al. [50]

The total energy input for the current switchgrass process, prior to enzyme hydrolysis, is the sum of the energy required for knife milling to 1 mm plus the energy required to heat the switchgrass from room temperature to 160 °C during 10 wt% ammonia treatment plus 5 min of attritor milling. The knife milling energy for milling switchgrass to 1 mm from Barakat et al. [21] is 0.828 MJ/kg (0.230 kW-h/kg). From measurements of the heat capacity of switchgrass [46] and the temperature dependence of the heat capacity for cellulose and lignin [47], including the enthalpy of the phase transition for lignin above 107 °C, the energy necessary to heat dry switchgrass from 25 to 160 °C was calculated to be 0.228 MJ/kg (0.0633 kW-h/kg). This calculation does not include the energy to heat the reactor, which is assumed to remain static at 160 °C. Finally the milling energy for 5 min of attritor milling (this work) was measured as 0.9576 MJ/kg (0.266 kW-h/kg). No energy was expended in drying the switchgrass, which was received dry (in most cases >91 % DM). The sum of these energy inputs is 2.013 MJ/kg (0.559 kW-h/kg). This sum corresponds to ~11 % of the energy content of the biomass (18.22 MJ/kg) [38, 39].

The gaseous ammonia plus fine milling process described here, with an overall energy efficiency of 0.280 kg/MJ for total reducing sugars (Table 5), compares favorably to the other processes, particularly considering that the calculations by Zhu and coworkers [20] for Steam explosion [48], Organosolv [49] and SPORL (Sulfite Pretreatment to Overcome Recalcitrance of Lignocellulose) [20, 50], conducted at 215, 170 and 180 °C, respectively, assume a 50 % reduction in the energy input through energy integration for these chemical pretreatments. Barakat and coworkers [22], provide the energy efficiency calculations for glucose only using wheat straw. The chemical treatments of Barakat et al. were all performed at 25 °C, followed by drying at 105 °C. Among these, the dry alkaline treatment (*L*/*S* = 0.2 w/w, 25 °C, 5 h, followed by drying) stands out with a high energy efficiency of 0.245 vs 0.0.154 kg/MJ for the gaseous ammonia plus attritor milling. However, these authors use, as the energy input for grinding and post-chemical treatment milling, the specific net energy consumption (the energy input with biomass present minus the energy input without biomass) which does not, therefore, include the energy necessary to operate the machinery independent of the biomass. The solids loadings for the enzymatic saccharifications are also 2 and 10 %, respectively, for the processes described by Zhu et al. and by Barakat et al. The solids loading used for the process described here in Table 5 was 25 %. The higher the solids loading, the lower the percent of theoretical recoverable sugar yield (see Fig. 5), thereby lowering the calculated energy efficiency. However, there is a downstream benefit to the high solids loading, as the sugars generated are far more concentrated, and the energy required to recover the fermentation product post-fermentation is much lower. The one other process in Table 5 applied to switchgrass, the microwave process by Karunanithy et al., is probably not competitive considering its very low energy efficiency.

## Conclusions

Ball milling of lignocellulosic biomass has a significant impact on the subsequent rate and yield of glucan and xylan hydrolysis during enzymatic saccharification. It does so by reducing particle size (increasing exposed surface area), by reducing the size of the cellulose crystalline domains and by producing, through compression and shear forces, homolytic bond cleavage of polysaccharide and lignin. In the absence of pretreatment, the energy necessary to generate these mechanochemical changes sufficient to produce commercially viable sugar yields upon saccharification, represents a significant fraction of the energy content of the biomass and is consequently not sustainable. However, by preceding ball milling with gaseous ammonia treatment, the milling energy required to produce high sugar yields from saccharified switchgrass is reduced, depending on the pretreatment conditions, four to eightfold relative to untreated biomass, making fine milling a potentially valuable accessory to lignocellulosic conversion to ethanol and other chemicals.

Attritor milling enormously accelerates the milling process relative to conventional ball milling—120 to 240-fold in the present case. From an energetic point of view, it also appears to be more energy efficient than other fine milling technologies that we have investigated—ball milling, pin milling, jet milling and air classifier milling. It also lowers the enzyme loading required for high saccharification yields.

A comparison of particle size and surface area and saccharification as a function of milling time shows a poor correlation except possibly early on in the milling process. In contrast, the enzymatic saccharification yield shows a negative linear dependence on the coherent domain size for non-pretreated switchgrass subjected to ball milling and a higher power dependence on coherent domain size when attritor milling is preceded by gaseous ammonia pretreatment. It is likely that, in the absence of pretreatment, milling produces dislocations in the biomass as well as bond cleavage of glucan, xylan and lignin that render the exposed ends of cellulose fibrils accessible to enzymatic saccharification. Preceding milling by high temperature gaseous ammonia pretreatment results in cleavage of the ester bonds between the lignin and the hemicellulose and the reorganization of the lignin, likely rendering the ends and the surfaces of the cellulose fibrils accessible to enzymatic digestion. One possible test of this conclusion might be that the endo-glucanases and xylanases as opposed to the exo-glucanases and xylanases, play a more significant role when milling is preceded by a high-temperature ammonia pretreatment than when it is not.

Finally, the moisture content present during the gaseous ammonia treatment has a significant impact on the saccharification yields. It is likely that the higher moisture in the presence of ammonia has enhanced the extent to which the acetyl and feruloyl ester bonds to xylan are cleaved. Such ester bond cleavage likely increases the ability of the xylanases present in the saccharification enzyme consortium to digest xylan as well as reducing the number of links between xylan and lignin. The reduced cross-linking of the latter structure would reduce physical barriers to enzyme diffusion and reduce the number of bonds that would need to be broken in the subsequent milling process. The favorable effect of moisture in the gaseous ammonia pretreatment would make even fall-harvested switchgrass a more attractive substrate for this process, given its higher moisture content. The ability as well to see a significant impact of room temperature incubation with gaseous ammonia on the energy required for fine milling would make it attractive to store harvested biomass in the presence of ammonia. By providing both chemical treatment and sterility, the presence of gaseous ammonia in storage could avoid a later high-temperature, energy consuming pretreatment step and prevent at the same time decay of the stored biomass.

The joining of the gaseous ammonia pretreatment to attritor milling is by no means the last word in the conversion of lignocellulosic biomass. The gaseous ammonia component of this process is attractive in that it uses a very low *L*/*S* ratio, minimizing the energy input for thermochemical treatment. The room temperature gaseous ammonia pretreatment has been much less explored. While already showing benefit in terms of lowering the energy required in subsequent milling, higher ammonia concentrations and/or longer incubation times may well accomplish under much milder conditions and at lower energy and capital cost what happens at higher temperature. There are also other milling technologies beyond those described here that may well be preferable in terms of energy efficiency and speed of milling. The attritor mill used here was operated in batch mode. A continuous milling technology would likely allow a higher throughput of milled biomass per unit time and a smaller capital investment provided that it also delivers the kind of impact and shear forces that make the attritor mill work.

## Methods

### Switchgrass

Three lots of switchgrass were used for these studies. UT-2, UT-4 and UT-5 were obtained from Genera Energy (Vonore, TN, USA). UT-2 and UT-4 were spring and fall-harvested, respectively. UT-4, the largest of the batches was knife-milled and pooled in two sub-batches with slightly different composition (UT-4 a and b, Table 1). UT-5, more closely resembles UT-4 than UT-2 in terms of carbohydrate and lignin content, both of which tend to be higher in spring-harvested switchgrass [39], implying that this batch is more likely fall-harvested. The carbohydrate and lignin compositions of these switchgrass lots are indicated in Table 1. All switchgrass samples, except where otherwise indicated, were knife-milled through a 1-mm screen in a Thomas-Wiley knife mill Model ED-5 (Arthur H. Thomas, Philadelphia, PA, USA).

### Saccharification conditions and sample preparation

The enzymatic saccharifications were performed in 50 or 100 mM Na citrate, pH 4.9 at a solids loading of 14 or 25 % wt/wt using Accellerase 1500 (Genencor, Palo Alto, CA, USA) 25–28 mg/g glucan, plus a 16.6 mg/g xylan cocktail of beta xylosidases from Fusarium or Accellerase DUET (Genencor, Palo Alto, CA, USA) at anywhere from 6.25 to 28 mg enzyme/g (glucan + xylan). At 14 % solids loading, 60 mg dry weight biomass was added to 364 μL Na citrate buffer including enzyme and saccharified on a rotary shaker (vials vertical) at 250 rpm at 47 °C in 6-mL glass vials containing two 5-mm glass beads. At 25 % solids loading, 75.8 mg dry weight biomass was added to 226 μL Na citrate buffer including enzyme and saccharified on a rotary shaker (vials vertical) at 250 rpm at 47 °C in 6-mL glass vials containing two 5-mm glass beads. Alternatively, 985 mg of dry weight samples were suspended in 2.94 mL Na citrate buffer including enzymes, placed in 6-mL glass vials containing two 5-mm glass beads and incubated at 47 °C on a rotating wheel (vials maintained in horizontal orientation) at ~2 rpm. Aliquots (10.3 μL) were taken at regular intervals, weighed, diluted to 792 μL with HPLC mobile phase (0.01 N H_2_SO_4_) and filtered through Spin-X centrifugal filters [0.22 or 0.45 μm, Nylon, Corning Life Sciences (Acton, MA, USA)] and loaded into HPLC vials for analysis.

To determine the concentration, if any, of soluble sugar oligomers, a 10.3 uL aliquot of the filtrate was diluted to 792 uL with 4 % H_2_SO_4_ and heated in a sealed vial in an autoclave for 1 h at 121 °C to hydrolyze any oligomers. Glucose, xylose and arabinose standards were treated similarly to correct for any sugar degradation. The autoclaved hydrolysate was then injected into the HPLC as above and the monomeric sugar concentrations compared before and after autoclaving. The difference was the concentration of soluble oligomers, expressed as monomeric sugars.

### Compositional analysis

The carbohydrate and acid-insoluble lignin compositions were determined using NREL procedure “Determination of Structural Carbohydrates and Lignin in Biomass” (Version 2006) [51].

### HPLC analysis

All sugar analyses were performed on a mixed Agilent 1100 and 1200 HPLC (Agilent Technologies, Wilmington, DE, USA) with a Biorad Aminex HPX-87H column plus guard column (Micro-Guard Cation H Cartridge) (Bio-Rad Laboratories, Hercules, CA, USA) using 0.01 N aqueous sulfuric acid as the eluent. The column was placed in a column oven at 60 °C and run at a flow rate of 0.6 mL/min. The refractive index detector was run at 55 °C.

### Ammonia pretreatment reactor

Ammonia pretreatment at elevated temperature was performed using a 5-L Littleford Day horizontal cylindrical pressure vessel (Littleford Day, Florence, KY, USA) modified to include a 1.5″ (3.8 cm) ball valve on the top of the reactor, which could be removed to charge the biomass. The reactor was equipped with two ports in the headspace, a 1.5″ ball valve on the bottom, various thermocouples, a relief valve, a pressure gauge, and a pressure transducer. The reactor contained a heat transfer-type impeller, which contained four blades for mixing solids vertically and horizontally. The impeller was rotated at approximately 40 rpm for all experiments. The high-temperature (150–160 °C) pretreatments with gaseous ammonia were performed at an ammonia loading of 10 wt% of biomass. A typical biomass per run charge was 500–600 g. The pretreatment runs were pooled to generate sufficient material for the milling trials. Because there was some temperature variation from run to run and during the runs, the temperature at which the treatments were performed ranged from 150 to 160 °C.

Extended room temperature incubations of biomass with ammonia were carried out at an ammonia loading of 20 wt% of biomass in a packed-bed reactor containing a steel filter basket (6.25″ in diameter, 29″ high) with solid sides and a 16 mesh screen at either end. The basket was placed inside a solid filter housing (8.25″ in diameter, 37.5′ high) and not stirred.

### Ball milling

Ball milling was carried out in 125-mL plastic milling bottles (5 cm diameter × 10 cm high) in which were contained 5 g of biomass and 200 g of ¼″ spherical stainless steel beads. Studies using steel beads of varying sizes showed the ¼″ beads to be optimal. The 125-mL plastic milling bottles were placed within a larger plastic bottle and oriented perpendicular to the long axis of the larger bottle such that, when the external bottle was placed on a U.S. Stoneware (Youngstown, OH, USA) model 755RMV jar mill, the milling bottles were rotated end over end, causing the beads to move from one end of the milling bottle to the other. The milling bottles were typically rotated at 83 rpm.

### Attritor milling

Attritor milling was carried out in batch mode in either an SD-1 or an S-1 attritor mill (Union Process, Akron OH) containing 500 g of switchgrass and 40 lbs of ¼″ spherical stainless steel beads. Milling in the SD-1 fitted with L-arms was carried out at an armature rotation speed of 516 rpm (2.14 hp) which delivers 0.958 MJ/kg biomass (0.266 kW-h/kg) of energy to the milling chamber in 5 min. The jacketed chamber was cooled by running tap water and the temperature after 5 min of milling was 27 °C. Given the very modest increase in temperature in 5 min, the cooling water is probably unnecessary. The top speed of the S-1 attritor is 400 rpm such that 8.3 min was required to deliver the same amount of energy as was delivered in 5 min for the SD-1 instrument at 516 rpm. No cooling water was used for the S-1 attritor mill.

### Surface area

Nitrogen adsorption/desorption measurements were performed at 77.3 °K on a Micromeritics (Norcross, GA, USA) ASAP model 2405 porosimeter. Samples were degassed at 150 °C for 12 h at <100 µmHg prior to data collection. Surface area measurements utilized a five-point adsorption isotherm collected over 0.05 to 0.20 *P*/*P*_0_ and analyzed via the BET method [52]. Pore volume distributions utilized a 27-point desorption isotherm and were analyzed via the BJH method [53]. *P* is the pressure of the gas above the sample (generally at liquid nitrogen boiling point temperature); *P*_0_ is the ideal gas pressure at the temperature of the sample being measured (typically around 760 Torr).

### Particle size

Particle size distribution measurements on ball-milled switchgrass were performed on a Horiba (Edison, NJ, USA) Laser Scattering Particle Size Distribution Analyzer LA-910 in deionized water. Particle size distribution measurements on Union Process attritor-milled switchgrass were performed on a Microtrac Inc. (Montgomeryville, PA, USA) S3500 laser diffraction particle analyzer.

### X-ray diffraction

X-ray diffraction data were obtained with a Philips X’PERT automated powder diffractometer, Model 3040. The diffractometer is equipped with automatic variable anti-scatter and divergence slits, X’Celerator RTMS detector, and Ni filter. The radiation is CuK(alpha) (45 kV, 40 mA). Data were collected at room temperature from 4° to 80° 2-theta; using a continuous scan with an equivalent step size of 0.02°; and a count time of 80 s per step in theta–theta geometry. Samples were packed into an aluminum sample holder and run as received, with no additional grinding. MDI/Jade software version 9.1 was used to remove sharp diffraction peaks from crystalline, inorganic contaminants and to convert the data to text format for further processing. Microsoft^®^ Excel^®^ is used for all additional processing.

## Abbreviations

KM: knife milled
DM: dry matter
*d*_50_: mass median diameter
*t*_1/2_: rise time—exponential half rise time
